# Transient selective aphasia in highly proficient bilinguals triggered by electrical stimulation of the left superior temporal gyrus

**DOI:** 10.1007/s00701-025-06508-5

**Published:** 2025-04-05

**Authors:** Ileana Quiñones, Sandra Gisbert-Muñoz, Garazi Bermudez, Iñigo Pomposo, Santiago Gil Robles, Manuel Carreiras, Lucia Amoruso

**Affiliations:** 1 Biogipuzkoa Health Research Institute, San Sebastian, Spain; 2 IKERBASQUE. Basque Foundation for Science, 48009 Bilbao, Spain; 3 ESIC Business and Marketing School, Valencia, Spain; 4 University of the Basque Country, UPV/EHU, 48940 Bilbao, Spain; 5 Department of Neurosurgery, Hospital Cruces, 48903 Bilbao, Spain; 6 BioCruces Research Institute, 48015 Bilbao, Spain; 7 Department of Neurosurgery, Hospital Quirónsalud, 28223 Madrid, Spain; 8 BCBL. Basque Center on Cognition, Brain, and Language, 20009 San Sebastian, Spain

**Keywords:** Bilingualism, Left superior temporal gyrus, Direct electrical stimulation

## Abstract

Are an individual’s first (L1) and second (L2) languages represented in shared or distinct brain territories? Using intraoperative electrical stimulation mapping (ESM) in two Basque-Spanish bilinguals with non-growing lesions—thus avoiding confounding effects of adaptive plasticity—this study identified distinct language representations within the left temporal lobe. Stimulation of posterior and anterior superior temporal gyri induced language-selective aphasias, whereas stimulation of the mid-temporal region and inferior fronto-occipital fasciculus produced naming errors without language specificity. These findings highlight both shared and distinct *loci* for L1 and L2, advancing our understanding of bilingual brain organization.

**Supplementary Information:**

The online version contains supplementary material available at 10.1007/s00701-025-06508-5.

## Introduction

Mapping the *locus* of both first (L1) and second (L2) language representation in the bilingual brain has long fascinated researchers. Central to this inquiry lies a longstanding yet unresolved question: Do languages inhabit shared or distinct neural territories? This debate has spurred extensive research [16], yielding mixed findings. Some studies show significant L1-L2 overlap [14, 17], suggesting a common neural landscape, while others [13] identify discrete brain regions dedicated to each language. Recently, intraoperative electrical stimulation mapping (ESM) during awake brain surgeries has proven effective for localizing language areas in bilingual patients [9, 12]. However, infiltrating lesions (e.g., brain tumors) often induce functional reorganization [7, 10], complicating efforts to pinpoint the “putative” neural signatures of L1 and L2.

Here, we examined language representation in two highly proficient Basque-Spanish bilinguals, with similar multilingual exposure and daily language-switching. Both harbored small benign left temporal lobe lesions. Unlike prior ESM studies involving bilinguals with infiltrating brain tumors [12, 19], our patients had “neurotypical” brains, free from tumor-driven reorganization. This unique opportunity allowed us to examine L1 and L2 production in early (< 3 years) bilinguals who used both languages interchangeably. Despite lesion location differences, surgical resection followed a similar approach, revealing overlapping regions of functional stimulation alongside the temporal lobe. By combining a bilingual picture-naming task with online ESM, we functionally mapped cortico-subcortical language pillars across L1 and L2, identifying structure-to-function relationships in neural tissue unaffected by adaptive plasticity.

## Materials and methods

### Clinical cases

**Fig. 1:**
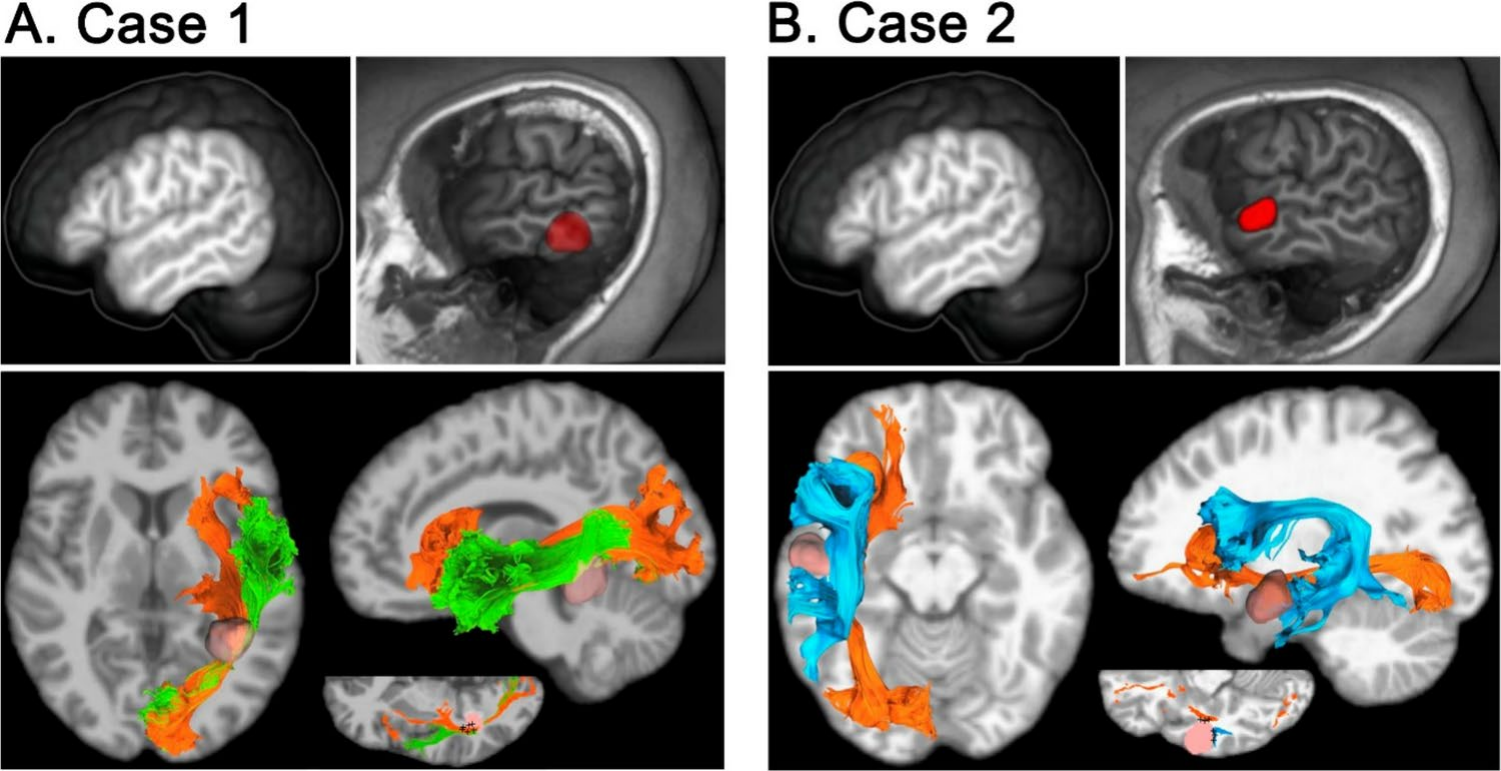
Imaging characterization of the two clinical cases using a 3 T Siemens Magnetom Prisma Fit scanner (Siemens AG, Erlangen, Germany). The upper left panel for each case displays the exposed cortical surface, revealing normal anatomy with no visible pathological signs. The upper right panel highlights the lesion location in red. The lower panel presents pre-surgical tractography reconstructions for both patients, illustrating the tracts adjacent to the lesion. Cavernoma resection in Case 1 followed a transcortical approach along the inferior temporal gyrus, involving the opening of the posterior part of the temporal horn of the lateral ventricle. For Case 2, DNET resection was conducted around the STG, between the superior and medial temporal gyri, up to the lateralmost wall of the sagittal striatum. Points associated with behavioral changes were marked as positive stimulation sites. For Case 1, the reconstructed tracts include the left IFOF and the Inferior Longitudinal Fasciculus (ILF). For Case 2, the tracts adjacent to the lesion are the IFOF and the Arcuate Fasciculus. Tracts are mapped onto T1-weighted images of each subject. The left side of the panel shows an axial view, while the right side provides a sagittal view. The axial view in the center of the panel includes black crosses indicating points where subcortical electrical stimulation was applied (see Supplementary Material for more details)

The study involved two highly proficient, right-handed individuals fluent in both Basque and Spanish, actively contributing to professional roles in a multilingual environment. Case 1 revolves around a 33-year-old woman who sought medical consultation for a persistent headache exacerbated by recumbency. A subsequent diagnosis revealed an incidental left temporomesial cavernoma. Case 2 involves a 23-year-old male diagnosed with a BRAF-mutated grade I dysembryoplastic neuroepithelial tumor (DNET), presenting with drug-resistant epilepsy five years prior (see Table 1 and Fig. 1).

**Table 1:** Characterization of the clinical cases

	**Case 1**	**Case 2**
Age	33	23
Biological sex	female	male
Educational level (years)	18	14
L1/L2 proficiency	100/100	100/100
L1/L2 age of acquisition	0/0	0/3
Daily L1/L2 exposure	60/40	60/40
Lesion volume (cm^3^)	3.59	2.37

Language proficiency was measured using the Basque, English, and Spanish picture-naming standardized tests [BEST] (Bruin et al., 2017). Daily exposure to both languages was estimated by asking them to rate the percentage of time spent per day listening, speaking, reading, and writing in Basque and Spanish. Ratings were averaged to obtain a self-rating composite score

### Intraoperative functional mapping

**Fig. 2:**
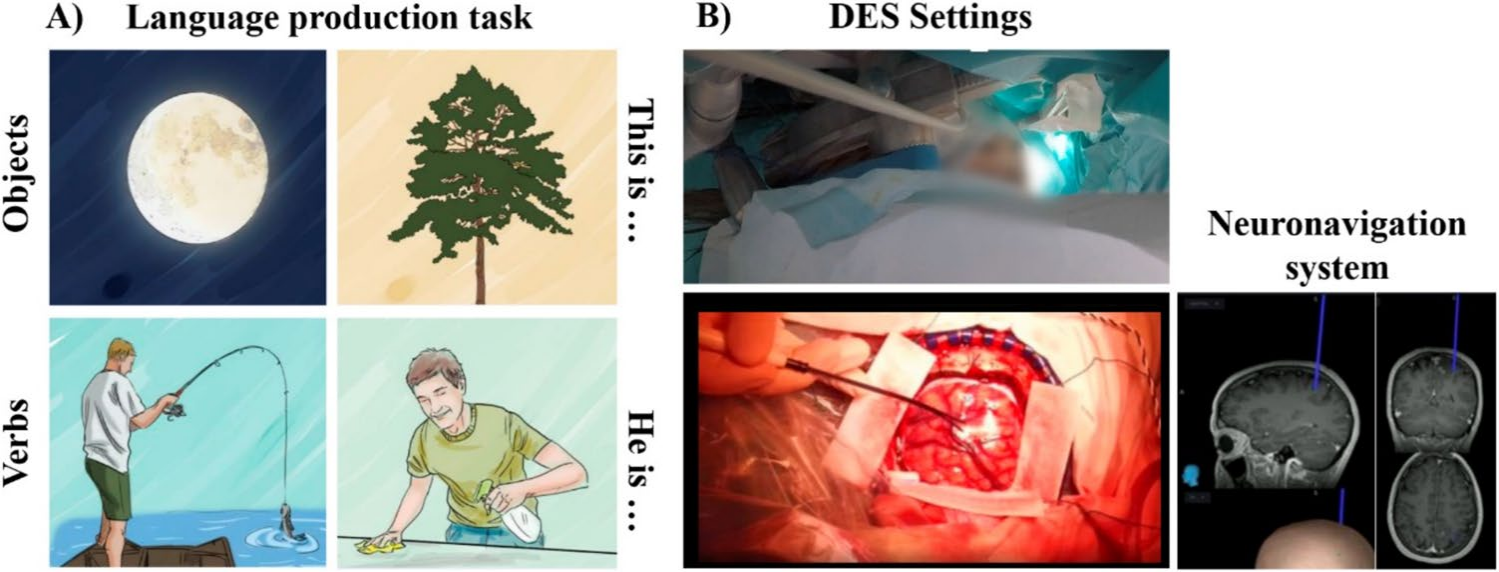
Experimental Protocol. (**A**) Examples of the images used during the language production task. These images were pre-validated to ensure hit levels above 85% [11]. (**B**) Recording process during intraoperative stimulation. Continuous audio-visual recordings of the surgical procedure were made while a rehabilitation physician monitored the patient's behavior. The procedure is guided by a neuronavigation system, which ensures precise localization of the stimulation coordinates throughout the surgery. A bipolar stimulation probe (Inomed, fork probe, 45 mm straight, ball tip diameter 2 mm, tip-to-tip distance 8 mm) was used at 2.5 mA. Only those sites where direct electrical stimulation-associated errors were replicated in at least 2 out of 3 non-consecutive trials were deemed as functional. The procedure lasted 4 h and 45 min for Case 1 and 2 h and 20 min for Case 2, with alternating periods of stimulation and rest. A total of 92 cortical and 39 subcortical points (131 in total), were respectively stimulated

Resective awake brain surgery was performed under the asleep-awake-asleep protocol for Case 1 and under conscious sedation with dexmedetomidine for Case 2. Intraoperative functional mapping was conducted using cortical and subcortical stimulation at 60 Hz, delivered with human-use certified intraoperative equipment (NimEclipse®, Medtronic). The entry point was determined through cortical stimulation, while subcortical stimulation was applied at specific functional limits. Positive functional responses were identified by the involuntary cessation (i.e., speech arrest) of counting during stimulation, as detailed in previous studies [15]. Different aspects of language processing (i.e., phonological, lexico-semantic, and syntactic) were assessed through a bilingual sentence completion task (see Fig. 2).

### Cluster analysis

During intraoperative stimulation, neurosurgeons mark sites as positive stimulation or eloquent areas if a behavioral change occurs two out of three times. However, due to the technique’s complexity, it is not feasible to quantify the replicability of the results in real-time. To address this issue and estimate the replicability of significant effects during stimulation both within and between individuals, a neighborhood-based clustering approach was employed.

## Results

### Intraoperative functional mapping

**Fig. 3:**
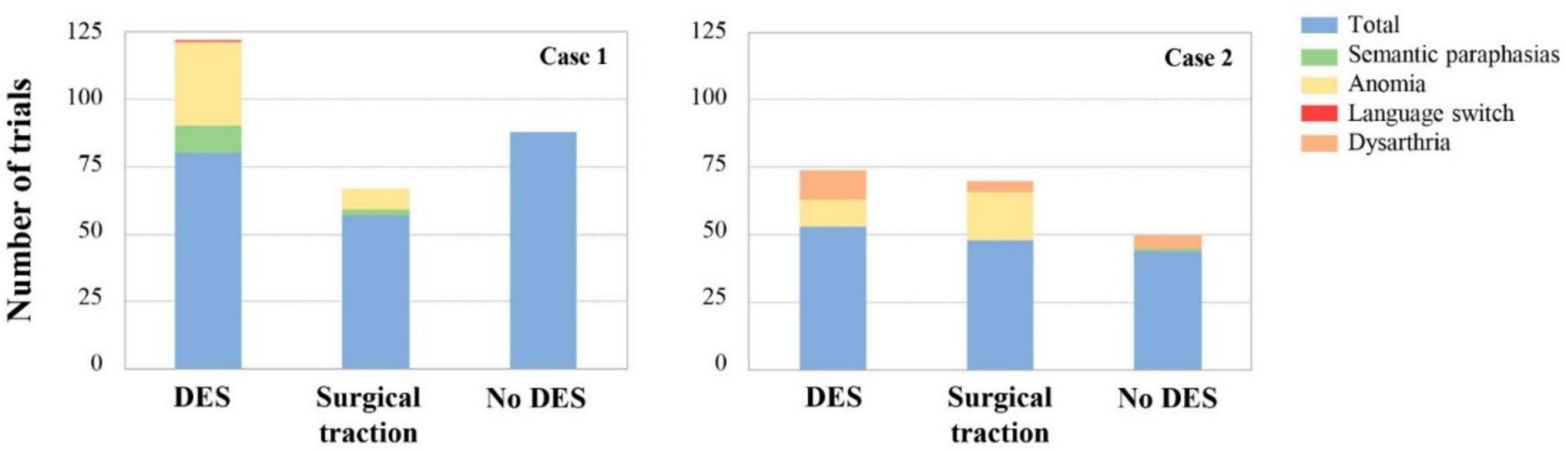
Patients'behavior during surgeries. Data from 216 trials, where patients were required to produce a sentence based on a depicted picture, were considered. These trials include both those conducted during direct electrical stimulation periods and those during lesion traction. **DES:** number of direct electrical stimulations performed during surgeries. Each stimulation is associated with an experimental trial where participants name images. **Surgical Traction:** experimental trials conducted during lesion removal. **No DES:** control trials where patients named images without stimulation. The total number of trials used varied for each patient, directly correlating with the duration of their surgeries

The intensity of the electrical stimulation was initially calibrated at the cortical level in the inferior ventral pre/motor cortex using a number counting task. In both clinical cases, positive responses (i.e., speech arrest) were evoked at the base of the premotor cortex. For bilingual language mapping, a total of 216 experimental trials were used from which positive stimulation points were identified and labeled based on the type of behavioral change observed. Specifically, four types of behavioral impairments became evident: the most frequent was anomia (77.22% of the positive trials), followed by dysarthria (12.66%), semantic paraphasias (8.86%), and one instance of a language switch error (1.27%), which could not be replicated (see Fig. 3).

**Fig. 4:**
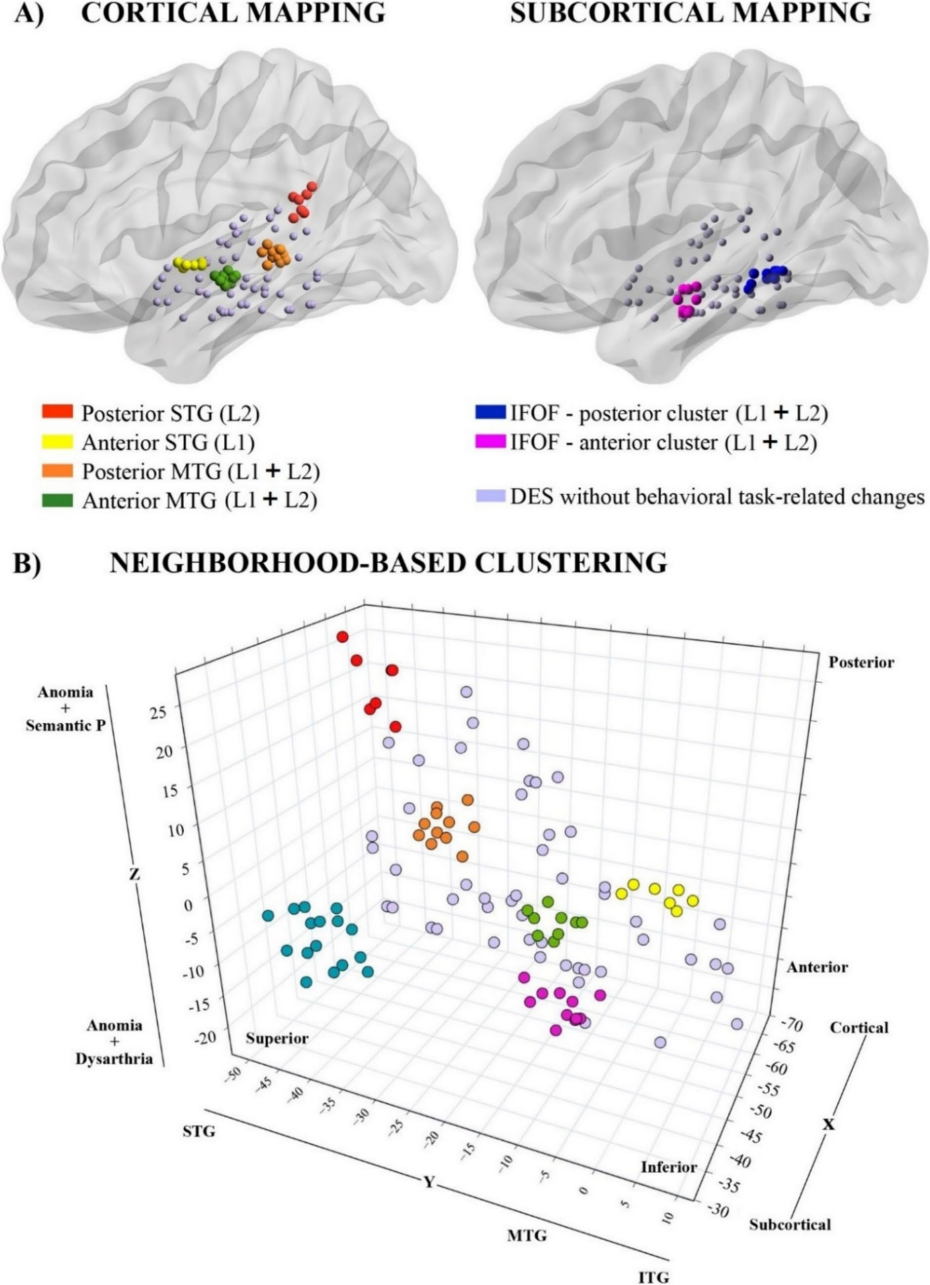
ESM of the left temporal cortex and adjacent white matter tracts. The cortical mapping includes the STG, MTG, and the inferior temporal gyrus. **A**) Positive and negative stimulation points are superimposed on a surface reconstructed from template BrainMesh_ICBM152Left.nv. The left panel shows cortical positive stimulation points, while the right panel displays subcortical positive stimulation points. The graphs were made using BrainNet Viewer, a brain network visualization tool designed by Xia, Wang and He [8]. **B**) Cluster analysis based on the stimulated coordinates (see Supplementary Materials for more details), resulting in six distinct clusters, each represented by a different color. At the cortical level, the algorithm identified four: posterior STG (cluster 1, MNI coordinates of the centroid [x, y, z]: − 59, − 45, 20), anterior STG (cluster 2, centroid: − 59, − 2, − 2), posterior MTG (cluster 3, centroid: − 59, − 35, 0), and anterior MTG (cluster 4, centroid: − 55 − 16 − 8). At the subcortical level, the cluster analysis grouped the data into only two clusters both located within the posterior segment of the left IFOF (clusters 5, centroid: − 39, − 43, − 8; and 6, centroid: − 45, − 13, − 13)

Specifically, in Case 1, three responsive regions were found. The first, located in the posterior segment of the left STG near the supramarginal gyrus, produced L2-specific anomias and semantic paraphasias (red dots in Fig. 4). The second, situated in the posterior part of the left MTG, captured anomias without language specificity (orange dots in Fig. 4). Subcortically, a third region was identified within the left IFOF (blue dots in Fig. 4), inducing anomias, semantic paraphasias, and one non-replicable language switch error. In Case 2, we identified three regions. The first, located in the anterior part of the STG, induced L1-specific anomias (yellow dots in Fig. 4). The second, at the anterior segment of the MTG, resulted in anomias and dysarthria in both languages (green dots in Fig. 4). The third, located in the anterior segment of the left IFOF, elicited anomias and dysarthria in both languages (violet dots in Fig. 4). Overall, in both patients, cortical stimulation of the medial segments of the STG and MTG did not produce any behavioral changes in either language. At the subcortical level, both patients exhibited similar behavioral responses inducing naming errors with no language-specific distinctions.

Coordinates of both cortical and subcortical positive stimulation points were further included in the neighborhood-based approach based on k-means clustering algorithm (see Table S1 and Fig. S1). At the cortical level, the algorithm identified four clusters largely overlapping with the regions identified during the surgery. However, at the subcortical level, although three distinct regions were identified during the surgery, the cluster analysis grouped the data into only two clusters both located within the posterior segment of the left IFOF (see Fig. 4).

### Postoperative clinical assessment

After surgery, the patient referred to as Case 1 experienced mild transient dysphasia in both L1 and L2, characterized by normal verbal comprehension but difficulties with lexical retrieval. However, the three-month postoperative neuropsychological assessment revealed no cognitive impairments. In Case 2, there was no neurological impairment following surgery, and no further seizures were observed during a five-year follow-up period.

## Discussion

In this study, we mapped L1 and L2 representation along cortical and subcortical regions of the left temporal lobe in two highly proficient bilinguals, revealing both shared and language-specific sites. ESM targeting the left STG—a key region involved in control monitoring during speech production—elicited selective aphasia-like symptoms in either L1 or L2, experimentally validating clinical instances of this condition. Conversely, stimulation of the left MTG and the IFOF—primarily involved in lexical-semantic processing—revealed common language sites. Although anomia was the most frequent error, we also found a functional anterior–posterior dissociation: posterior stimulation produced semantic paraphasias, whereas anterior stimulation caused dysarthria. These findings are discussed in detail below.^1^

### Evidence for language-specific microcircuits within the left STG

Since Wernicke’s seminal work in 1974, the left STG has been recognized as central to speech perception and production [4]. In our study, ESM over the left STG resulted in aphasic-like symptoms (i.e., word-retrieval difficulties) during a sentence completion task requiring overt picture naming. This aligns with evidence from aphasic patients linking anomia to atrophy in both anterior and posterior segments of the left STG [1]. Likewise, previous ESM studies in tumor patients [6, 20], show that anomia can be elicited via stimulation of the left STG, reinforcing its critical role in speech production. A key finding, however, was the localization of distinct sites for L1 and L2 errors within the STG. Contrary to accounts of overlapping L1–L2 representation in highly proficient individuals [14, 17], our results provide direct ESM evidence of language-specific effects. This suggests that, even in early, highly proficient bilinguals, the network supporting language production exhibits language-dependent variations at the microcircuit level in the left STG (see Fig. S2). Further, it underscores how MRI, though invaluable for mapping broad neural networks, may miss fine-grained functional organization within language areas (see [5] for evidence supporting the microcellular arrangement of the STG).

### Evidence for overlapping L1 and L2 representation along the left MTG

Two positive-stimulation clusters emerged along the left MTG, where ESM triggered transient anomia with no language specificity. Interestingly, a recent meta-analysis of lesion-symptom mapping studies [18] highlights this region's critical involvement in the early stage of word production, namely during lexico-semantic retrieval. Furthermore, evidence shows that, even after controlling for visual and motor-speech deficits in chronic stroke patients, only the left mid-posterior MTG and its adjacent white matter area were significantly linked to performance in picture naming [2]. Additionally, our findings in the left MTG mirrored the gradient observed along the STG, with posterior MTG stimulation producing semantic paraphasias and anterior stimulation resulting in dysarthria. While posterior MTG are involved in lexico-semantic aspects of word retrieval, more anterior regions contribute to sentence-level processing and integrating lexico-semantic and grammatical information [3]. Given that our sentence-completion task required number and gender agreement, disrupting this combinatorial process likely explains the observed effects. Overall, these findings underscore the left MTG’s pivotal role in integrating and producing fluent speech, irrespective of the language in use.

## Conclusions

These findings highlight the coexistence of shared and distinct regions for language production, depending on the linguistic processes engaged. Clinically, this underscores the importance of personalized care that accounts for both linguistic and neural uniqueness. Understanding bilingual brain organization, both pre- and intraoperatively, is crucial for fully preserving all spoken languages.

## Supplementary Information

Below is the link to the electronic supplementary material.

Supplementary file1 (DOCX 477 KB)

## Data Availability

Availability of data and material: The data presented in this study as well as the cognitive tasks and experimental stimuli used during the surgery are available on request from the corresponding author. The data are not publicly available due to the data-sharing policies of the different institutions involved. Code availability: Not applicable.

## Footnotes

1 See also the Supplementary Material for additional considerations.
