# Supplementary material for: Transient selective aphasia in highly proficient bilinguals triggered by electrical stimulation of the left superior temporal gyrus
Source: Acta Neurochir (Wien). 2025 Apr 5;167(1):99. doi: 10.1007/s00701-025-06508-5 (PMC11972172; doi:10.1007/s00701-025-06508-5)
Supplement: Supplementary file 1 — Supplementary file1 (DOCX 477 KB) [file 701_2025_6508_MOESM1_ESM.docx]

# **SUPLEMENTARY INFORMATION**

**Supplementary Materials and Methods**

*Presurgical Radiological Study.* Both patients underwent a comprehensive MRI session using a 3T Siemens Magnetom Prisma Fit scanner (Siemens AG, Erlangen, Germany). High-resolution T1- and T2-weighted images were acquired through a 3D ultrafast gradient echo MPRAGE pulse sequence using a 64-channel head coil with the following acquisition parameters for T1: 176 contiguous sagittal slices, voxel resolution 1x1x1 mm3, Repetition Time (RT) = 2530 ms, Echo Time (ET) = 2.36 ms, Image columns = 256, Image rows = 256, flip angle (Flip) = 7˚; and for T2: 176 contiguous sagittal slices, voxel resolution 1x1x1 mm3, RT = 3390 ms, ET = 389 ms, Image columns = 204, Image rows = 256, Flip = 120˚. The origin of the T1/T2 weighted images was set to the anterior commissure and co-registered using the T1 as reference. The imaging protocol revealed T1-weighted hypointense and T2-weighted hyperintense cortical lesions, with annular enhancement post-gadolinium injection in the case of DNET. This T1 and T2 signal intensity change was confirmed with a relative hypointense signal on fluid attenuation inversion recovery (FLAIR) MR sequences. T1 and T2 images were jointly utilized to derive a transformation matrix essential for normalizing the images to the MNI space. This involved using the segmentation function implemented in the Computational Anatomy Toolbox (version 12) (<https://neuro-jena.github.io/cat/>), which classifies each voxel into distinct tissue classes (e.g., GM, WM, and CSF) and assigns corresponding labels based on a generative model, thereby delineating specific anatomical structures. The segmentation output facilitated the transition between individual space and MNI space, allowing for the integration of spatial coordinates representing stimulation points across patients into a unified brain template to run the cluster analysis.

In addition, diffusion-weighted images (DWI) were acquired using a 2D EPI diffusion sequence (TE = 99 ms, TR = 9300 ms, slice thickness = 1.8 mm, in-plane resolution = 1.79688 mm). A diffusion tensor scheme was used, and a total of 64 diffusion sampling directions were acquired. The processing of DWI was done using DSI-Studio (<https://dsi-studio.labsolver.org/>). The b-value was 1500 s/mm^2^. The b-table was checked by an automatic quality control routine to ensure its accuracy (*1*). The restricted diffusion was quantified using restricted diffusion imaging (*2*). The diffusion data were reconstructed using generalized q-sampling imaging (GQI) (*3*) with a diffusion sampling length ratio of 1.25 and thread count = 16, using cerebrospinal fluid calibration for better accuracy. GQI is a model-free method that calculates the orientational distribution of the density of diffusing water, termed the spin distribution function, by solving for an analytic relation using simple matrix multiplication (*3*). Fibers were tracked with deterministic tracking using the RK4 method (*4*), with FA threshold = 0.08, Otsu threshold = 0.6, trilinear interpolation, a step size of 0.25mm, and a max turning angle of 60 degrees. A max fiber length of 300 mm was used, and a max fiber count of 10,000 tracts. Since the program may run forever if 10,000 tracts are not found, we used a max of 100,000 seeds. After the fiber extraction, automatic topology-informed pruning (TIP) was completed five times, to remove false fiber trajectories (*5*).

This technique enables the mapping of white matter functional boundaries at the subcortical level, allowing for the examination of underlying white matter tracts that mediate information flow within the temporal lobe, with a particular focus on the left inferior fronto-occipital fascicle (IFOF). The IFOF is the longest association bundle in the human brain, uniquely connecting occipital, temporal, parietal, and frontal areas, making it crucial for mapping visual information into meaning (*6*). Indeed, previous studies involving both healthy individuals and patients with brain lesions have shown that the IFOF is implicated in semantic processing during picture naming (*7, 8*), with decreased performance correlating with IFOF integrity (*9*). Additionally, intraoperative stimulation of multiple portions of the IFOF disrupts semantic processing during picture naming (*10-12*), leading to different types of speech errors such as anomias and semantic paraphasias (e.g., saying "lion" instead of "tiger"). Overall, the left temporal regions and their underlying white matter tracts are involved in key aspects of language production (*13-18*), making them core structures for investigating bilingual representation during naming.

*Awake brain surgery.* Resective awake brain surgery was performed under the asleep-awake-asleep protocol for Case 1 and under conscious sedation with dexmedetomidine for Case 2. Real-time online monitoring of language functions guided the surgery, aiming to identify functionally significant structures (positive response sites) and optimize volume resection while minimizing the risk of postoperative functional impairments. In both cases a left temporal craniotomy was performed, exposing temporal areas, including the temporoparietal junction and the ventral part of the motor area. The latter was used for the calibration of the electrical stimulation.

*Intraoperative Functional Mapping.* During awake craniotomy, electrical stimulation mapping (ESM) involves the neurosurgeon applying direct electrical stimulation to a specific brain region employing a bipolar electrode while the patient engages in language tasks (e.g., picture naming). If the patient shows behavioral changes (e.g., speech arrest, anomia, or paraphasia) in two out of three non-consecutive stimulation trials, the area is deemed functional and preserved from resection. The use of this technique in clinical settings offers a unique and rare opportunity to investigate real-time structure-function mapping with fine-grained spatial resolution (*19*). Intraoperative functional mapping was conducted using cortical and subcortical stimulation. The entry point was determined through cortical stimulation, while subcortical stimulation was applied at specific functional limits. During the resection process, motor function assessment involved two main approaches. Firstly, muscle contractions in the face or limbs contralateral to the lesion were monitored. Secondly, alterations in motor tasks, such as simultaneous flexion-extension of the hand, arm, and forearm contralateral to the lesion, were observed. Spontaneous language production was evaluated using a counting paradigm (series from 0 to 10). Positive functional responses were identified by the involuntary cessation (i.e., speech arrest) of counting during stimulation, as detailed in previous studies (*20*). Different aspects of language processing (i.e., phonological, lexico-semantic, and syntactic) were assessed through sentence completion tasks in Spanish and Basque. These tasks allow the distinction between different types of errors (*21*) such as phonological or semantic paraphasias (e.g., substitutions of phonemes such as *“pike”* instead of *“pipe”* or substitution of words that are semantically related to the intended one, such as *'table'* instead of *'chair'*), pure anomias (i.e., impaired recall of words caused by difficulties during lexical access), dysarthria (i.e., improperly articulated speech), alexia (i.e., inability to read), or reading errors (i.e., letter omissions or substitutions). Behavioral responses during stimulation were closely monitored by a rehabilitation physician.

*Sentence completion task*. We utilized a subset of 40 images from the multilingual picture naming test “MULTIMAP” (*22*). The task comprised two blocks of object/action naming, conducted in Basque and Spanish respectively. The selected target words were matched between languages in terms of frequency, number of orthographic neighbors, and length (5-8 characters). Stimuli were also controlled for visual complexity, familiarity, and naming agreement (> 85%). In each block, participants were presented with an image of an object or an action accompanied by an introductory phrase (see Fig. 2 for additional details). Participants were required to complete the sentences with the corresponding noun or verb depicted in the image, ensuring agreement in number and gender. Each trial began with a white screen featuring a black fixation cross for 1 second, followed by the display of the image for 4 seconds. An auditory cue was emitted 500 milliseconds before the onset of each stimulus to synchronize the presentation with the stimulation. The beginning of the subsequent trial was controlled by the physician interacting with the patient. To mitigate interference effects between languages, evaluations were conducted sequentially in separate blocks, one language at a time. Participants' responses, including accuracy and reaction time per trial, were recorded. The stimuli presentation, along with the MATLAB script and its compiled version, are accessible for use at <https://git.bcbl.eu/sgisbert/multimap2>. Importantly, patients' ability to perform the task was evaluated before the surgery (i.e., baseline) and items that elicited incorrect responses were excluded from the intraoperative mapping to minimize the risk of false positives.

Cortical and subcortical mapping were conducted using direct electrical stimulation at 60 Hz, delivered with human-use certified intraoperative equipment (NimEclipse®, Medtronic). A bipolar stimulation probe (Inomed, fork probe, 45 mm straight, ball tip diameter 2 mm, tip-to-tip distance 8 mm) was used at 2.5 mA. Cavernoma resection in Case 1 followed a transcortical approach along the inferior temporal gyrus, involving the opening of the posterior part of the temporal horn of the lateral ventricle. For Case 2, DNET resection was conducted around the STG, between the superior and medial temporal gyri, up to the lateralmost wall of the sagittal striatum. The procedure lasted 4 hours and 45 minutes for Case 1 and 2 hours and 20 minutes for Case 2, with alternating periods of stimulation and rest. A total of 92 cortical and 39 subcortical points (131 in total), were respectively stimulated. Points associated with behavioral changes were marked as positive stimulation sites. Only those sites where direct electrical stimulation-associated errors were replicated in at least 2 out of 3 non-consecutive trials were deemed as functional.

*Cluster analysis.* During intraoperative stimulation, neurosurgeons mark sites as positive stimulation or eloquent areas if a behavioral change occurs two out of three times. However, due to the technique's complexity, it is not feasible to quantify the replicability of the results in real-time. To address this issue and estimate the replicability of significant effects during stimulation both within and between individuals, a neighborhood-based clustering approach was employed. This method combines the positive and negative stimulation sites from both patients, enabling the assessment of the distribution patterns of spatial coordinates and the behavioral changes induced by electrical stimulation. Initially, Elbow method optimization was used for determining the optimal number of clusters. The number of clusters was iterated from 1 to 10, with the cluster configuration yielding the highest silhouette score selected as optimal (see Table S1). The k-means clustering information reported includes cluster size, explained proportion of within-cluster heterogeneity, and the centroid (i.e., [x, y, z] MNI coordinates). The Euclidean distance (i.e., root sum-of-squares of differences) between the centroids of the two resulting clusters was also calculated and reported (see Table S2). ESM results are reported by combining data from both patients.

| **Table S1.** Neighborhood-Based Approach based on K-Means Clustering Algorithm. | | | | | | | | | | | | | |  |  |  |  |  |  |  |
| --- | --- | --- | --- | --- | --- | --- | --- | --- | --- | --- | --- | --- | --- | --- | --- | --- | --- | --- | --- | --- |
| Clusters | N | R² | AIC | | | BIC | | | Silhouette | | | | | |  |  |  |  |  |  |
| 6 | 133 | 0.832 | 102.540 | | | 154.570 | | | 0.450 | | | | | |  |  |  |  |  |  |
| *Note.* The model was optimized with respect to the *BIC* value (BIC = 154.57, AIC = 102.54, R2 = 0.83, Silhouette = 0.45). | | | | | | | | | | | | | |  |  |  |  |  |  |  |
| **Table S2.** Cluster Information | | | | | | | | | | | | | | | | | | | | |
| Cluster | | | | | 1 | | | 2 | | | 3 | | 4 | | | | 5 | | 6 | |
| Size | | | |  | 24 | |  | 24 | |  | 30 |  | 23 | | |  | 20 |  | 12 |  |
| Explained proportion within-cluster heterogeneity | | | |  | 0.265 | |  | 0.202 | |  | 0.077 |  | 0.196 | | |  | 0.172 |  | 0.089 |  |
| Within sum of squares | | | |  | 17.620 | |  | 13.449 | |  | 5.133 |  | 13.016 | | |  | 11.414 |  | 5.911 |  |

**Figure S1.** Elbow Method Plot


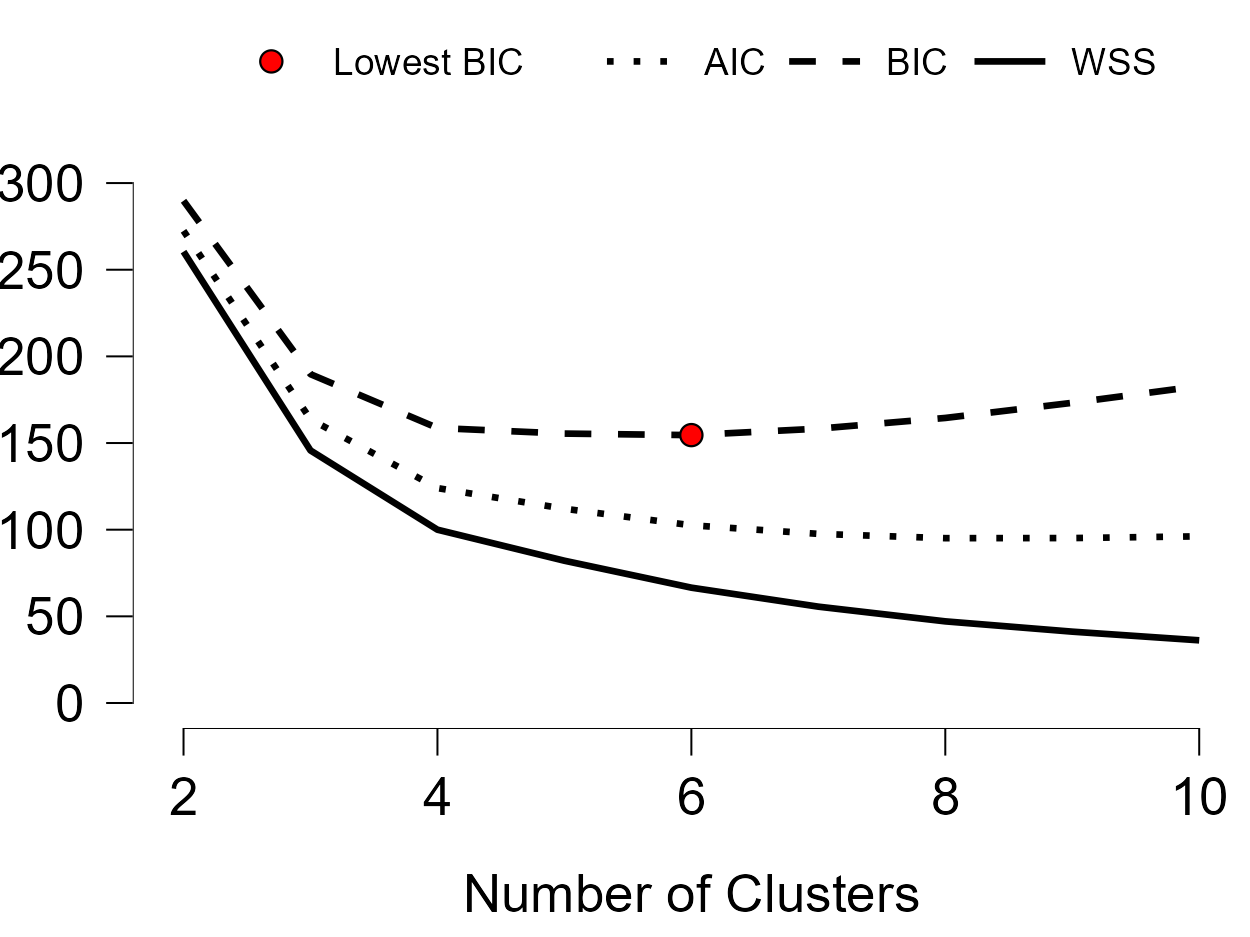


**Supplementary Theoretical Considerations**

*Evidence for language-specific microcircuits within the left STG.* From Wernicke’s seminal work in 1974 to modern clinical aphasiology, the left STG has been recognized for its crucial role in both speech perception and production (*23*). In our study, ESM over the left STG resulted in aphasic-like symptoms (i.e., difficulties in word retrieval) during a sentence completion task requiring the overt naming of pictures. This aligns with prior evidence from aphasic patients (*24*) showing that anomia is linked to atrophy in both anterior and posterior segments of the left STG (*25*). Similarly, studies using ESM in patients with brain tumors (*11, 21, 26, 27*), show that anomia can be elicited via stimulation of the left STG, further highlighting its essential role in speech production.

A critical finding, however, was the observation of distinct sites for L1 and L2 errors along the STG. In contrast to evidence suggesting overlapping L1 and L2 representation in the bilingual brain of highly proficient individuals (*28-30*), our study provides direct ESM evidence to the contrary. The network supporting language production in early, highly proficient bilinguals, exhibits language-dependent variations, at the level of the left STG. Interestingly, this result corroborates a recent fMRI study from our laboratory, which used the same task and a comparable population of healthy Basque-Spanish bilinguals (*31*). That study, employing machine learning techniques, assessed whether the functional organization of the language network could predict the language a person was speaking. Strikingly, it was found that the left STG was the top-ranked region showing maximum discrimination between languages during naming. However, the methodological approach of that study considered the STG as a single functional region. Thus, present ESM evidence opens new avenues of research on the bilingual brain, highlighting that, while MRI studies provide insights into brain networks at a macroscopic level, they may overlook the organization at the microcircuit level within functional areas (see *32 for evidence supporting the microcellular arrengment of the STG*). Specifically, stimulation of the posterior segment of the left STG caused L2-selective errors, while stimulation of the anterior segment resulted in L1-selective deficits.

A similar pattern of ESM during awake brain surgery has been previously observed in bilingual patients with brain tumors (*33*), exhibiting both language-specific and overlapping sites along temporoparietal areas, including the posterior segment of the left STG. Interestingly, despite the variability in language proficiency and AoA among these patients, some of them (e.g., patients 2, 3, 10, and 12) showed language-specific sites along the left posterior STG. In line with these results, a recently published study conducted a longitudinal follow-up using ESM on patients with gliomas who had undergone multiple awake surgeries (*34*). This study demonstrated that the STG is among the most susceptible regions to neuroplastic reorganization. Intracortical mapping of the STG revealed variable results across surgeries, highlighting the dynamic nature of neural adaptation in this region. However, generalizing these results proves challenging due to the bias induced by neuroplasticity mechanisms: does the representation of L1 and L2 diverge due to compensatory mechanisms differentially affecting each language? Here, by examining the representation of L1 and L2 within the left STG in "neurotypical" cases, we provide direct evidence for the notion of bilingual language specialization within this region, offering insights that are unaffected by neuroplastic changes associated with the evolution of oncological pathologies.

Despite mounting evidence for STG's involvement in language production and comprehension, its precise role within the language network remains unclear, as highlighted by a recent meta-analysis (*35*). Various functions have been attributed to it. On the one hand, it has been proposed that this region is involved in accessing the phonological form of words (i.e., lexical phonology) (*36-38*) and in translating auditory to motor templates for accurate word articulation (*39, 40*). On the other hand, neuroanatomical models of language production place it at the center of speech control mechanisms, orchestrating the interface between transmitters, receivers, and articulators (*15-18, 41-43*). Although evidence is limited, electrocorticographic studies further support this latter view, suggesting a microcellular arrangement in the STG that allows conflict monitoring at various levels (*44*). This finding aligns with the microcircuit organization observed in our study, where a clear functional posterior-anterior distinction within the STG was found.

Disentangling our experimental design, the difference in task performance between L1 and L2 in highly proficient bilinguals lies not in accessing the lexical-semantic features of the target word, which would be shared (*45*), but in accessing grammatical, phonological, and articulatory features, as well as the combinatorial mechanisms that generate correct responses. For example, viewing an image of a "tree" would activate largely overlapping lexical-semantic representations for L1 and L2 words (e.g., "árbol" in Spanish and "zuhaitz" in Basque), but their distinct grammatical, acoustic, and motor patterns would likely activate specialized cortical circuits. This interpretation is further supported by the fact that no language specificity was observed along the MTG (see below), which is linked to early lexico-semantic access during speech production (*46*).

Furthermore, different types of errors were found along this posterior-anterior gradient within the STG: semantic paraphasias were triggered at posterior sites, while dysarthria was induced in more anterior regions. To shed light on this functional dissociation, we explored structure-to-function relationships based on fMRI meta-analytic data using Neurosynth^^[[1]](#footnote-1)^^ (<https://compose.neurosynth.org/>, see Fig. S2). The two STG centroids identified by the cluster-based analysis were used to track their connectivity profiles. Interestingly, the posterior STG centroid was connected with key left-lateralized language hubs involved in controlled lexico-semantic retrieval, such as the left inferior frontal gyrus (IFG) and the posterior MTG (*47*), which can explain the observation of semantic errors. Indeed, there is evidence showing that stimulation over the posterior STG can lead to both semantic and phonological paraphasias depending on whether targeting local connectivity along ventral or dorsal pathways, respectively (*48*). Conversely, the anterior centroid in which dysarthria-related stimulation sites were more evident was connected to a cortico-subcortical bilateral network subserving articulatory motor planning and control monitoring during word production (*36, 49-53*), engaging the supplementary motor area, prefrontal structures as well as the basal ganglia.

The presence of language-specific microcircuits within the left STG may reflect the speech control loop previously proposed (*15-18, 41-43*). Assuming that the posterior part of the STG monitors processes related to the access and retrieval of lexical-semantic information, it is plausible that the non-dominant language imposes a greater neural cost than the dominant one. Previous studies indicate that inhibiting information associated with the dominant language is necessary to retrieve information in L2. This cognitive overload could account for the effects observed in the posterior segment. Such specialization likely facilitates the precise control needed to avoid cross-language effects during speech production, as both languages are co-activated even when only one is required for communication (*54, 55*). It has been suggested that in highly proficient bilinguals, this coactivation is accompanied by inhibition or selection mechanisms, which could explain the effects observed in the present study (*56*).


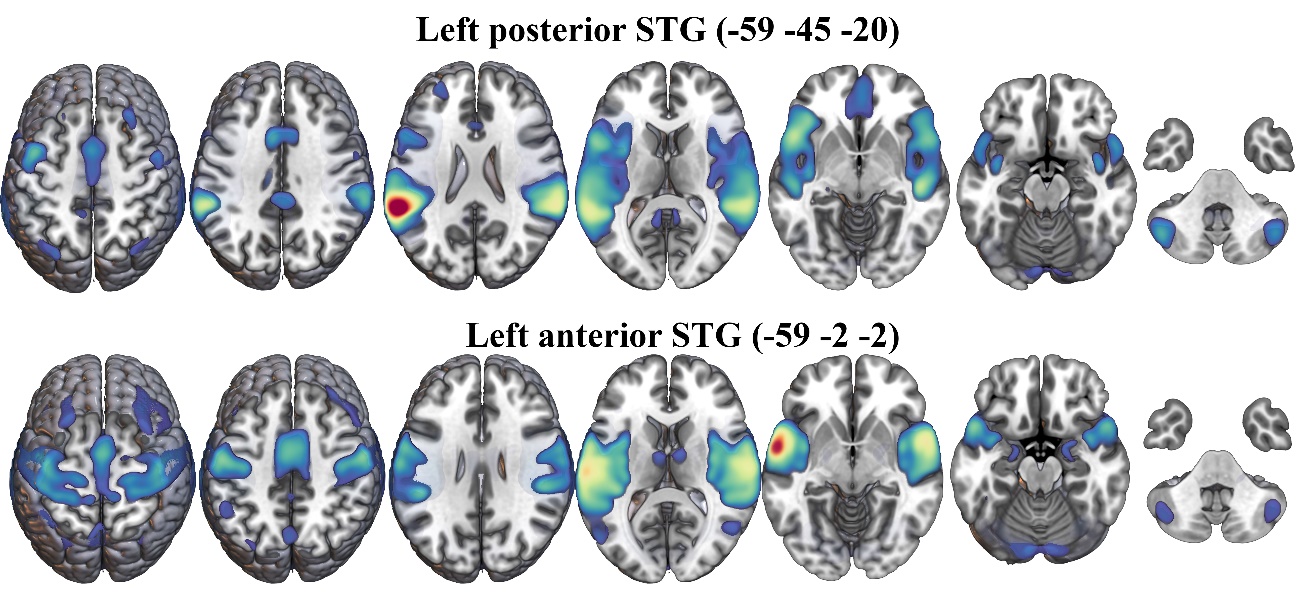


**Figure S2.** Resting-state functional connectivity for two seed regions (left posterior and anterior STG) in a sample of 1,000 subjects estimated using Neurosynth (<https://neurosynth.org/>), a platform for large-scale, automated synthesis of fMRI data. It takes thousands of published articles reporting the results of fMRI studies. To reduce the blurring of signals across cerebro-cerebellar and cerebro-striatal boundaries, fMRI signals from adjacent cerebral cortex were regressed from the cerebellum and striatum (see Yeo et al (2011), Buckner et al (2011), and Choi et al (2012) for methodological details).

In contrast, the anterior segment of the STG may respond to differences in later stages of processing. Control mechanisms at the anterior level monitor combinatorial operations, integrating the extracted linguistic information with contextual cues. Previous research has demonstrated that integrating information from the dominant language is more neurally demanding than from L2, even in cases of high proficiency (see *57 for concordant behavioral results*). This evidence suggests that, in our non-dominant language, we remain at a superficial level of context analysis, which could explain the observed effects associated exclusively with L1 (*58-62*). Thus, in the context of bilingualism, the STG appears to regulate language output by continuously controlling competition between L1 and L2 at different levels. It would be highly valuable to count with ESM or electrocorticography data contrasting the effects of inhibiting STG and IFG functionality —the other key region involved in speech control (*15-18, 41-43*). This would enable the development of a more comprehensive theoretical model of bilingual control mechanisms related to language production.

*Evidence for overlapping L1 and L2 representations along the left MTG.* Two clusters of positive stimulation were identified along the left MTG, where the electrical stimulation triggered transient anomia without language specificity. Interestingly, a recent meta-analysis of lesion-symptom mapping studies (*46*) highlights this region's critical involvement in the early stage of word production, namely during lexico-semantic retrieval. Furthermore, evidence shows that, after controlling for deficits in visual recognition and motor speech dysfluency in chronic stroke patients, the only brain regions that remained significantly related to performance in the Boston Naming Test were located in the left mid-posterior MTG and the adjacent white matter area (*63*). Similar findings have been reported in brain tumor patients, reinforcing its critical role in lexical-semantic access stages (*11*). Additionally, several studies targeting the posterior MTG with inhibitory transcranial magnetic stimulation support its causal involvement in controlled aspects of word retrieval during picture naming (*64-66*).

It has been suggested that, with increased dual-language experience, non-native speakers tend to process their L2 similarly to their L1, particularly at the lexico-semantic level (*45*). Accordingly, several neuroimaging studies have shown overlapping brain activity in highly proficient bilinguals during L1 and L2 picture naming (*67-69*), within a time frame consistent with lexical-semantic access (*49*). Furthermore, in a recent transcranial magnetic stimulation study, we applied inhibitory stimulation over the left posterior MTG in a group of highly proficient bilinguals and observed that while this region was causally involved in L1 and L2 word retrieval, responses did not differ between languages (*65*). Overall, our finding of similar L1 and L2 responses along the MTG further supports the idea of shared representations at the lexico-semantic level in the bilingual brain.

Additionally, our findings in the left MTG mirrored the gradient observed along the STG, with posterior MTG stimulation producing semantic paraphasias and anterior stimulation resulting in dysarthria. While more posterior regions of the MTG are involved in lexico-semantic aspects of word retrieval, more anterior regions have been implicated in sentence-level processing and the integration of lexico-semantic and grammatical information (*70*). Given that the task used here involved a sentence completion that had to agree in number and gender, a possible interpretation is the disruption of this combinatorial process. Previous evidence shows that damage to the underlying connectivity between the anterior MTG and the IFG (pars orbitalis; BA 47) results in speech fluency impairments, highlighting its key role in lexically based aspects of connected speech (*71*). Furthermore, using TMS for preoperative noninvasive language mapping in mid-to-anterior MTG regions results in positive stimulation sites associated with dysarthria and hesitation (*72*). Thus, these findings collectively underscore the importance of the left MTG in the integration and production of fluent speech, irrespectively of the language in use.

*Subcortical mapping of the left IFOF does not differentiate between languages.* The standard procedure for awake surgery involves using subcortical stimulation to delineate the functional boundaries of the affected area. Tract identification is based on the cognitive-functional changes induced by electrical stimulation. In this study, we introduced preoperative tractography to accurately identify tracts adjacent to the explored area, aiding in the distinction between the left arcuate, IFOF, and ILF during surgery. Given the role of these tracts in oral language production, transient inhibition of this connectivity causes changes that are undetectable using a picture naming task. In this case, preoperative tractography (Fig. 1) provided essential information for identifying the IFOF as the target tract for subcortical stimulation, offering crucial methodological support to the neurosurgery team. In our study, while we successfully mapped the IFOF and established subcortical functional boundaries during resection, no language dissociation was observed along this tract. Studies using ESM in brain tumor patients suggest that the left IFOF supports visual lexical-semantic access, playing a crucial role in mapping visual information into meaning during picture naming (*6, 11, 73, 74*). However, empirical evidence regarding potential distinctions between languages at the subcortical level remains limited *(96)*. Moreover, to the best of our knowledge, and in line with our results, no study has reported language dissociations in bilinguals within the left IFOF.

Previous evidence indicates that lexical-semantic processes are supported cortically by the MTG and subcortically by the IFOF (*75-77*). Models of bilingualism suggest that in highly proficient bilinguals, L1 and L2 representations converge at the lexical-semantic level (*45, 75*). Our findings extend this understanding by showing that the overlap of L1 and L2 representations not only includes the gray matter cortical territory of the left MTG but also the surrounding white matter tract associated with the left IFOF. Additionally, the finding of common sites for both languages along the MTG and language-specific regions in the STG is consistent with evidence showing that while STG functional connectivity is mediated by the arcuate fasciculus, MTG connectivity is mediated by the IFOF (*77*). These findings support the hypothesis that early bilingualism primarily impacts the structural connectivity of brain regions involved in speech control mechanisms rather than those involved in lexico-semantic retrieval (*76, 78*). Indeed, subcortical stimulation of the arcuate fasciculus triggered language-specific errors in bilinguals, including anomia, phonemic paraphasias, and dysarthria (*79*). Lastly, a recent MRI study found that early bilinguals, like those tested here, exhibit structural differences along the arcuate fasciculus but not in the IFOF (*80*), providing further support to the selective structural impact of experiential factors.

Interestingly, it has been shown that stimulation of the left IFOF can elicit a wide spectrum of naming errors (*21*), including anomia, perseverations, semantic paraphasias, comprehension errors, and morphosyntactic impairments. This diversity has led to hypotheses proposing two functionally distinct branches within the left IFOF: a ventral lexical-semantic branch linking parietal and anterior temporal regions, terminating in the IFG (pars orbitalis), and a dorsal branch associated with combinatorial functions, connecting occipito-temporal structures with prefrontal regions, passing through the pars triangularis and opercularis within the left IFG (*76*). By demonstrating a functional dissociation between the posterior-inferior and posterior-superior segments of the left IFOF—producing semantic paraphasias and dysarthria, respectively—we provide direct evidence for the existence of distinct branches within this region supporting distinct aspects of speech production. Overall, this dual-branch model offers a more nuanced understanding of the IFOF's role in language processing and explains the diversity in naming errors reported both in our study and in the literature.

**References**

1. K. G. Schilling *et al.*, Challenges in diffusion MRI tractography–Lessons learned from international benchmark competitions. *Magnetic resonance imaging* **57**, 194-209 (2019).

2. F. C. Yeh, L. Liu, T. K. Hitchens, Y. L. Wu, Mapping immune cell infiltration using restricted diffusion MRI. *Magnetic resonance in medicine* **77**, 603-612 (2017).

3. F.-C. Yeh, V. J. Wedeen, W.-Y. I. Tseng, Generalized q-sampling imaging. *IEEE transactions on medical imaging* **29**, 1626-1635 (2010).

4. P. J. Basser, S. Pajevic, C. Pierpaoli, J. Duda, A. Aldroubi, In vivo fiber tractography using DT‐MRI data. *Magnetic resonance in medicine* **44**, 625-632 (2000).

5. H. S. Wang, N. Y. Wang, F. C. Yeh, Specifying the diffusion MRI connectome in Chinese-speaking children with developmental dyslexia and auditory processing deficits. *Pediatrics and neonatology* **60**, 297-304 (2019).

6. S. Sarubbo *et al.*, Towards a functional atlas of human white matter. *Human brain mapping* **36**, 3117-3136 (2015).

7. G. I. de Zubicaray, S. E. Rose, K. L. McMahon, The structure and connectivity of semantic memory in the healthy older adult brain. *NeuroImage* **54**, 1488-1494 (2011).

8. W. D. Hula *et al.*, Structural white matter connectometry of word production in aphasia: an observational study. *Brain : a journal of neurology* **143**, 2532-2544 (2020).

9. J. C. Griffis, R. Nenert, J. B. Allendorfer, J. P. Szaflarski, Linking left hemispheric tissue preservation to fMRI language task activation in chronic stroke patients. *Cortex; a journal devoted to the study of the nervous system and behavior* **96**, 1-18 (2017).

10. J. Sierpowska *et al.*, White-matter pathways and semantic processing: intrasurgical and lesion-symptom mapping evidence. *NeuroImage. Clinical* **22**, 101704 (2019).

11. S. Sarubbo *et al.*, Mapping critical cortical hubs and white matter pathways by direct electrical stimulation: an original functional atlas of the human brain. *NeuroImage* **205**, 116237 (2020).

12. H. Duffau, S. Moritz-Gasser, E. Mandonnet, A re-examination of neural basis of language processing: proposal of a dynamic hodotopical model from data provided by brain stimulation mapping during picture naming. *Brain and language* **131**, 1-10 (2014).

13. P. Hagoort, Nodes and networks in the neural architecture for language: Broca's region and beyond. *Current opinion in neurobiology* **28C**, 136-141 (2014).

14. G. Hickok, D. Poeppel, The cortical organization of speech processing. *Nature Reviews Neuroscience* **8**, 393-402 (2007).

15. F. H. Guenther, *Neural control of speech*. (Mit Press, 2016).

16. F. H. Guenther, Cortical interactions underlying the production of speech sounds. *Journal of communication disorders* **39**, 350-365 (2006).

17. F. H. Guenther, J. S. Perkell, B. Maassen, R. Kent, H. Peters, A neural model of speech production and its application to studies of the role of auditory feedback in speech. *Speech motor control in normal and disordered speech*, 29-49 (2004).

18. H. E. Miller, F. H. Guenther, Modelling speech motor programming and apraxia of speech in the DIVA/GODIVA neurocomputational framework. *Aphasiology* **35**, 424-441 (2021).

19. Y. Wang, A. Metoki, K. H. Alm, I. R. Olson, White matter pathways and social cognition. *Neuroscience and biobehavioral reviews* **90**, 350-370 (2018).

20. E. Mandonnet, S. Sarubbo, H. Duffau, Proposal of an optimized strategy for intraoperative testing of speech and language during awake mapping. *Neurosurgical review* **40**, 29-35 (2017).

21. E. Collee *et al.*, Localization patterns of speech and language errors during awake brain surgery: a systematic review. *Neurosurgical review* **46**, 38 (2023).

22. S. Gisbert-Munoz *et al.*, MULTIMAP: Multilingual picture naming test for mapping eloquent areas during awake surgeries. *Behav Res Methods* **53**, 918-927 (2021).

23. B. R. Buchsbaum, M. D'Esposito, The search for the phonological store: from loop to convolution. *Journal of cognitive neuroscience* **20**, 762-778 (2008).

24. D. R. Akhmadullina, R. N. Konovalov, Y. A. Shpilyukova, E. Y. Fedotova, S. N. Illarioshkin, Anomia: Deciphering Functional Neuroanatomy in Primary Progressive Aphasia Variants. *Brain sciences* **13**, (2023).

25. F. Piras, P. Marangolo, Noun-verb naming in aphasia: a voxel-based lesion-symptom mapping study. *Neuroreport* **18**, 1455-1458 (2007).

26. J. Lu *et al.*, Functional maps of direct electrical stimulation-induced speech arrest and anomia: a multicentre retrospective study. *Brain : a journal of neurology* **144**, 2541-2553 (2021).

27. H. Duffau, Awake Surgery for Left Posterior Insular Low-Grade Glioma Through the Parietorolandic Operculum: The Need to Preserve the Functional Connectivity. A Case Series. *Frontiers in surgery* **8**, 824003 (2021).

28. A. E. Hernandez, M. Dapretto, J. Mazziotta, S. Bookheimer, Language switching and language representation in Spanish-English bilinguals: an fMRI study. *NeuroImage* **14**, 510-520 (2001).

29. D. Perani *et al.*, The bilingual brain. Proficiency and age of acquisition of the second language. *Brain : a journal of neurology* **121 ( Pt 10)**, 1841-1852 (1998).

30. D. Klein, B. Milner, R. J. Zatorre, E. Meyer, A. C. Evans, The neural substrates underlying word generation: a bilingual functional-imaging study. *Proceedings of the National Academy of Sciences of the United States of America* **92**, 2899-2903 (1995).

31. I. Quinones *et al.*, Unveiling the neuroplastic capacity of the bilingual brain: Insights from healthy and pathological individuals. *Brain, Structure and Function* **in press.**, (2024).

32. A. M. Chan *et al.*, Speech-specific tuning of neurons in human superior temporal gyrus. *Cerebral Cortex* **24**, 2679-2693 (2014).

33. F. E. Roux, M. Tremoulet, Organization of language areas in bilingual patients: a cortical stimulation study. *Journal of neurosurgery* **97**, 857-864 (2002).

34. S. Ng *et al.*, Intraoperative functional remapping unveils evolving patterns of cortical plasticity. *Brain : a journal of neurology* **146**, 3088-3100 (2023).

35. S. Meekings, S. K. Scott, Error in the superior temporal gyrus? A systematic review and activation likelihood estimation meta-analysis of speech production studies. *Journal of Cognitive Neuroscience* **33**, 422-444 (2021).

36. P. Indefrey, W. J. Levelt, The spatial and temporal signatures of word production components. *Cognition* **92**, 101-144 (2004).

37. W. J. Levelt, A. Roelofs, A. S. Meyer, A theory of lexical access in speech production. *The Behavioral and brain sciences* **22**, 1-38; discussion 38-75 (1999).

38. W. W. Graves, T. J. Grabowski, S. Mehta, P. Gupta, The left posterior superior temporal gyrus participates specifically in accessing lexical phonology. *Journal of cognitive neuroscience* **20**, 1698-1710 (2008).

39. G. Hickok, D. Poeppel, Dorsal and ventral streams: a framework for understanding aspects of the functional anatomy of language. *Cognition* **92**, 67-99 (2004).

40. J. E. Warren, R. J. Wise, J. D. Warren, Sounds do-able: auditory-motor transformations and the posterior temporal plane. *Trends in neurosciences* **28**, 636-643 (2005).

41. E. Golfinopoulos, J. A. Tourville, F. H. Guenther, The integration of large-scale neural network modeling and functional brain imaging in speech motor control. *NeuroImage* **52**, 862-874 (2010).

42. F. H. Guenther, in *ICPhS*. (2015).

43. E. Runnqvist, in *Language Production*. (Routledge, 2023), pp. 168-190.

44. M. Ozker, I. M. Schepers, J. F. Magnotti, D. Yoshor, M. S. Beauchamp, A double dissociation between anterior and posterior superior temporal gyrus for processing audiovisual speech demonstrated by electrocorticography. *Journal of cognitive neuroscience* **29**, 1044-1060 (2017).

45. J. Abutalebi, D. Green, Bilingual language production: The neurocognition of language representation and control. *Journal of Neurolinguistics* **20**, 242–275 (2007).

46. V. Piai, D. Eikelboom, Brain Areas Critical for Picture Naming: A Systematic Review and Meta-Analysis of Lesion-Symptom Mapping Studies. *Neurobiol Lang (Camb)* **4**, 280-296 (2023).

47. C. Whitney, M. Kirk, J. O'Sullivan, M. A. Lambon Ralph, E. Jefferies, The neural organization of semantic control: TMS evidence for a distributed network in left inferior frontal and posterior middle temporal gyrus. *Cerebral cortex* **21**, 1066-1075 (2011).

48. E. T. McKinnon *et al.*, Types of naming errors in chronic post-stroke aphasia are dissociated by dual stream axonal loss. *Scientific reports* **8**, 14352 (2018).

49. P. Indefrey, The spatial and temporal signatures of word production components: a critical update. *Frontiers in psychology* **2**, 255 (2011).

50. F. X. Alario, H. Chainay, S. Lehericy, L. Cohen, The role of the supplementary motor area (SMA) in word production. *Brain research* **1076**, 129-143 (2006).

51. M. Liljestrom, C. Stevenson, J. Kujala, R. Salmelin, Task- and stimulus-related cortical networks in language production: Exploring similarity of MEG- and fMRI-derived functional connectivity. *NeuroImage* **120**, 75-87 (2015).

52. S. Ries, N. Janssen, B. Burle, F. X. Alario, Response-locked brain dynamics of word production. *PloS one* **8**, e58197 (2013).

53. R. D. Kent, J. F. Kent, G. Weismer, J. R. Duffy, What dysarthrias can tell us about the neural control of speech. *Journal of Phonetics* **28**, 273-302 (2000).

54. S. Villameriel, B. Costello, M. Giezen, M. Carreiras, Cross-modal and cross-language activation in bilinguals reveals lexical competition even when words or signs are unheard or unseen. *Proceedings of the National Academy of Sciences* **119**, e2203906119 (2022).

55. S. C. Bobb, K. Von Holzen, J. Mayor, N. Mani, M. Carreiras, Co-activation of the L2 during L1 auditory processing: An ERP cross-modal priming study. *Brain and language* **203**, 104739 (2020).

56. A. Costa, M. Santesteban, I. Ivanova, How do highly proficient bilinguals control their lexicalization process? Inhibitory and language-specific selection mechanisms are both functional. *Journal of experimental psychology. Learning, memory, and cognition* **32**, 1057-1074 (2006).

57. X. Cai, Y. Yin, Q. Zhang, A cross-language study on feedforward and feedback control of voice intensity in Chinese–English bilinguals. *Appl. Psycholinguist* **41**, 771-795 (2020).

58. E. Runnqvist, T. H. Gollan, A. Costa, V. S. Ferreira, A disadvantage in bilingual sentence production modulated by syntactic frequency and similarity across languages. *Cognition* **129**, 256-263 (2013).

59. E. Runnqvist, K. Strijkers, J. Sadat, A. Costa, On the temporal and functional origin of l2 disadvantages in speech production: a critical review. *Frontiers in psychology* **2**, 379 (2011).

60. K. Strijkers, C. Baus, E. Runnqvist, I. Fitzpatrick, A. Costa, The temporal dynamics of first versus second language production. *Brain and language* **127**, 6-11 (2013).

61. A. L. Beatty-Martínez *et al.*, Interactional context mediates the consequences of bilingualism for language and cognition. *Journal of Experimental Psychology: Learning, Memory, and Cognition* **46**, 1022 (2020).

62. K. Jankowiak, Current trends in electrophysiological research on bilingual language processing. *Language and Linguistics Compass* **15**, e12436 (2021).

63. J. V. Baldo, A. Arevalo, J. P. Patterson, N. F. Dronkers, Grey and white matter correlates of picture naming: evidence from a voxel-based lesion analysis of the Boston Naming Test. *Cortex; a journal devoted to the study of the nervous system and behavior* **49**, 658-667 (2013).

64. K. Krieger-Redwood, E. Jefferies, TMS interferes with lexical-semantic retrieval in left inferior frontal gyrus and posterior middle temporal gyrus: Evidence from cyclical picture naming. *Neuropsychologia* **64**, 24-32 (2014).

65. P. Timofeeva *et al.*, Switching off: disruptive TMS reveals distinct contributions of the posterior middle temporal gyrus and angular gyrus to bilingual speech production. *Cerebral cortex* **34**, (2024).

66. E. Jefferies, The neural basis of semantic cognition: converging evidence from neuropsychology, neuroimaging and TMS. *Cortex; a journal devoted to the study of the nervous system and behavior* **49**, 611-625 (2013).

67. S. Geng *et al.*, Oscillatory dynamics underlying noun and verb production in highly proficient bilinguals. *Scientific reports* **12**, 764 (2022).

68. P. Timofeeva *et al.*, Behavioral and oscillatory signatures of switch costs in highly proficient bilinguals. *Scientific reports* **13**, 7725 (2023).

69. E. Van de Putte, W. De Baene, M. Brass, W. Duyck, Neural overlap of L1 and L2 semantic representations in speech: A decoding approach. *NeuroImage* **162**, 106-116 (2017).

70. J. Brennan, L. Pylkkänen, The time-course and spatial distribution of brain activity associated with sentence processing. *NeuroImage* **60**, 1139-1148 (2012).

71. J. Fridriksson, D. Guo, P. Fillmore, A. Holland, C. Rorden, Damage to the anterior arcuate fasciculus predicts non-fluent speech production in aphasia. *Brain : a journal of neurology* **136**, 3451-3460 (2013).

72. I. Bahrend *et al.*, Incidence and linguistic quality of speech errors: a comparison of preoperative transcranial magnetic stimulation and intraoperative direct cortex stimulation. *Journal of neurosurgery* **134**, 1409-1418 (2020).

73. H. Duffau *et al.*, New insights into the anatomo-functional connectivity of the semantic system: a study using cortico-subcortical electrostimulations. *Brain : a journal of neurology* **128**, 797-810 (2005).

74. F. Almairac, G. Herbet, S. Moritz-Gasser, N. M. de Champfleur, H. Duffau, The left inferior fronto-occipital fasciculus subserves language semantics: a multilevel lesion study. *Brain structure & function* **220**, 1983-1995 (2015).

75. J. Jarret *et al.*, Functional network and structural connections involved in picture naming. *Brain and language* **231**, 105146 (2022).

76. E. Shekari, N. Nozari, A narrative review of the anatomy and function of the white matter tracts in language production and comprehension. *Frontiers in human neuroscience* **17**, 1139292 (2023).

77. M. Catani, V. Bambini, A model for social communication and language evolution and development (SCALED). *Current opinion in neurobiology* **28**, 165-171 (2014).

78. F. J. Liegeois *et al.*, Dorsal language stream anomalies in an inherited speech disorder. *Brain : a journal of neurology* **142**, 966-977 (2019).

79. L. Bello *et al.*, Intraoperative language localization in multilingual patients with gliomas. *Neurosurgery* **59**, 115-125; discussion 115-125 (2006).

80. S. Hamalainen, V. Sairanen, A. Leminen, M. Lehtonen, Bilingualism modulates the white matter structure of language-related pathways. *NeuroImage* **152**, 249-257 (2017).

1. A tool designed to meta-analyze structure-function relationships based on fMRI data (i.e., activation maps, and resting state functional connectivity [↑](#footnote-ref-1)
